# Cough-Induced Multiple, Bilateral, Asymmetrical Rib Fractures in a Scoliosis Patient: A Case Report

**DOI:** 10.7759/cureus.49251

**Published:** 2023-11-22

**Authors:** Zsuzsánna Incze-Bartha, Aurelio Pio Russo, Ylenia Pastorello, Vlad Vunvulea, Nicolae Stanciu, Mattia Mattarocci, Lóránd Dénes

**Affiliations:** 1 Department of Anatomy and Embryology, George Emil Palade University of Medicine, Pharmacy, Science, and Technology of Târgu Mureș, Târgu Mureș, ROU; 2 Department of Orthopedic Surgery and Traumatology, Dr. Fogolyán Kristóf Emergency County Hospital, Sfântu Gheorghe, ROU; 3 Faculty of Medicine, George Emil Palade University of Medicine, Pharmacy, Science, and Technology of Târgu Mureș, Târgu Mureș, ROU; 4 Department of Radiology, Emergency County Hospital Târgu Mureș, Târgu Mureș, ROU; 5 Department of Orthopedic Surgery and Traumatology, Emergency County Hospital Târgu Mureș, Târgu Mureș, ROU; 6 Department of Aesthetic Medicine, The Ghanem Clinic, London, GBR

**Keywords:** thoracic pain, scoliosis, multiple rib fractures, rib deformities, cough

## Abstract

Cough-induced rib fractures represent infrequent complications of strenuous and prolonged coughing, mostly provoked by respiratory tract infections, with localized chest pain being the most indicative component of the clinical picture. This paper reports a case of a 27-year-old female patient who presented with four cough-induced rib fractures following the contraction of an upper respiratory tract infection. The unique character of this case is provided by the young age of the patient, the presence of multiple and bilaterally located rib fractures, and the absence of predisposing factors related to her bone physiology. Furthermore, three of the four fractures were revealed on the left side, where a scoliotic sinistro-convex thoracic curvature is described. Following conservative treatment, the patient experienced a complete resolution of symptoms and favorable clinical outcomes. Even in the seemingly low-risk category, the diagnosis of cough-induced rib fractures should be taken into consideration, and their correlation to pre-existing rib deformities, such as the ones secondary to scoliosis, should be thoroughly investigated.

## Introduction

Rib fractures are defined as disruption of the costal bone integrity more commonly following car accidents, falls, sports-related impacts, or, in general, traumatic events. Stress fractures of the ribs represent an infrequent occurrence, with athletes being the most affected cohort, as a consequence of a repetitive submaximal musculoskeletal loading during training [1].

Coughing serves as a vital reflexive mechanism facilitating the effective clearance of bronchial secretions; however, its prolonged and vigorous paroxysms induce strenuous contraction of the respiratory muscles acting on the bony thorax, therefore accounting as a rare etiology of stress rib fractures [1,2].

This paper reports a case of a 27-year-old female patient presenting with multiple bilateral cough-induced rib fractures associated with an upper respiratory tract infection.

## Case presentation

A 27-year-old female patient presented to her family doctor with a one-week history of progressively intensifying cough attacks, voice hoarseness, and rhinorrhea, treated with paracetamol, nonsteroidal anti-inflammatory drugs (NSAIDs), throat lozenges, and decongestants. The aforementioned symptoms were accompanied by moderate left hypochondriac pain, starting from day five. No symptom relief was provided by the medications, therefore antibiotic treatment with clarithromycin (500 mg twice per day for seven days) and low dose betamethasone (5 mg per day for five days) were prescribed, with a diagnosis of acute upper respiratory tract infection and intercostal muscle strain due to persistent cough attacks.

New-onset, insidious, right upper quadrant pain was experienced at the beginning of the second week. The pain, now present bilaterally, displayed a sharp, localized character, with a marked increase in intensity upon exertion, laughing, and coughing episodes, which sporadically triggered emesis. A “cracking” sensation was described predominantly on the left side during these activities, with a concomitant small, hard thoracic swelling, revealed during clinical examination in right lateral decubitus. Naproxen and myorelaxants mildly attenuated the symptoms, although side sleeping remained significantly uncomfortable. To investigate the underlying cause of localized pain, the patient was referred for a chest roentgenogram.

**Figure 1:**
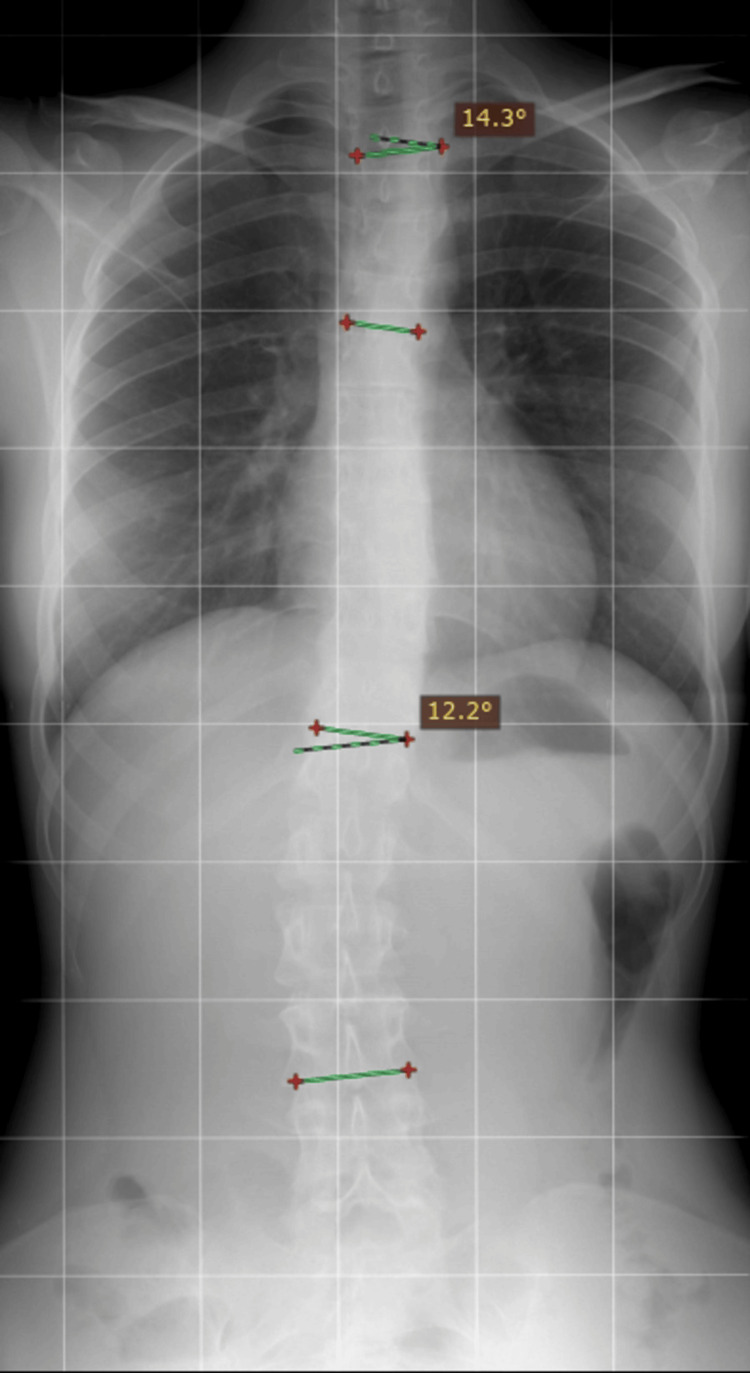
Last X-ray performed for scoliosis monitoring. Sinistro-convex thoracic and dextro-convex lumbar curvatures with Cobb angles of 14.3 and 12.2 degrees, respectively (RadiAnt DICOM Viewer). Costal integrity, consistent with the pre-infection findings, is displayed.

It is worth mentioning the presence of mild scoliosis with a combined character, diagnosed during adolescence, for which the patient undergoes regular X-ray assessments, as shown in her last check-up, several months prior to the contraction of the respiratory infection discussed here (Figure 1).

**Figure 2:**
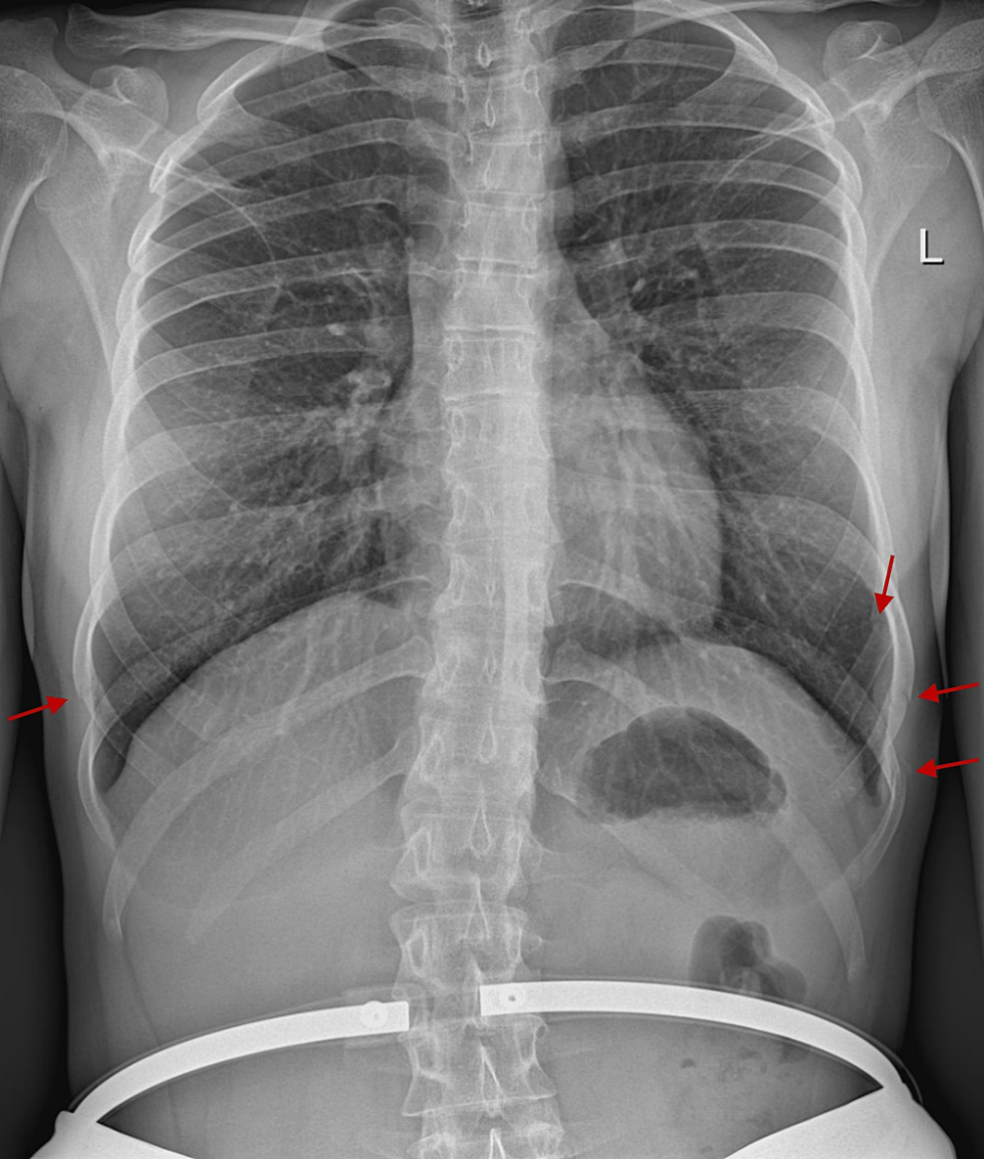
Chest X-ray performed four weeks after onset of symptoms. Red arrows point to four rib fractures, namely, the left eighth, ninth, and tenth ribs, and the right ninth rib.

The prescribed chest X-ray posteroanterior view highlighted three rib fractures on the left side, namely, ribs nine, 10, and 11, and one rib fracture on the right side, specifically rib nine (Figure 2).

Laboratory investigations, aimed at excluding underlying etiologies for pathological costal fractures, resulted within the normal range, except for a minor decrease in vitamin D serum levels (26.71 ng/mL; reference values: 30-100 ng/mL). Management of this case consisted of conservative treatment and vitamin D and calcium supplementation.

**Figure 3:**
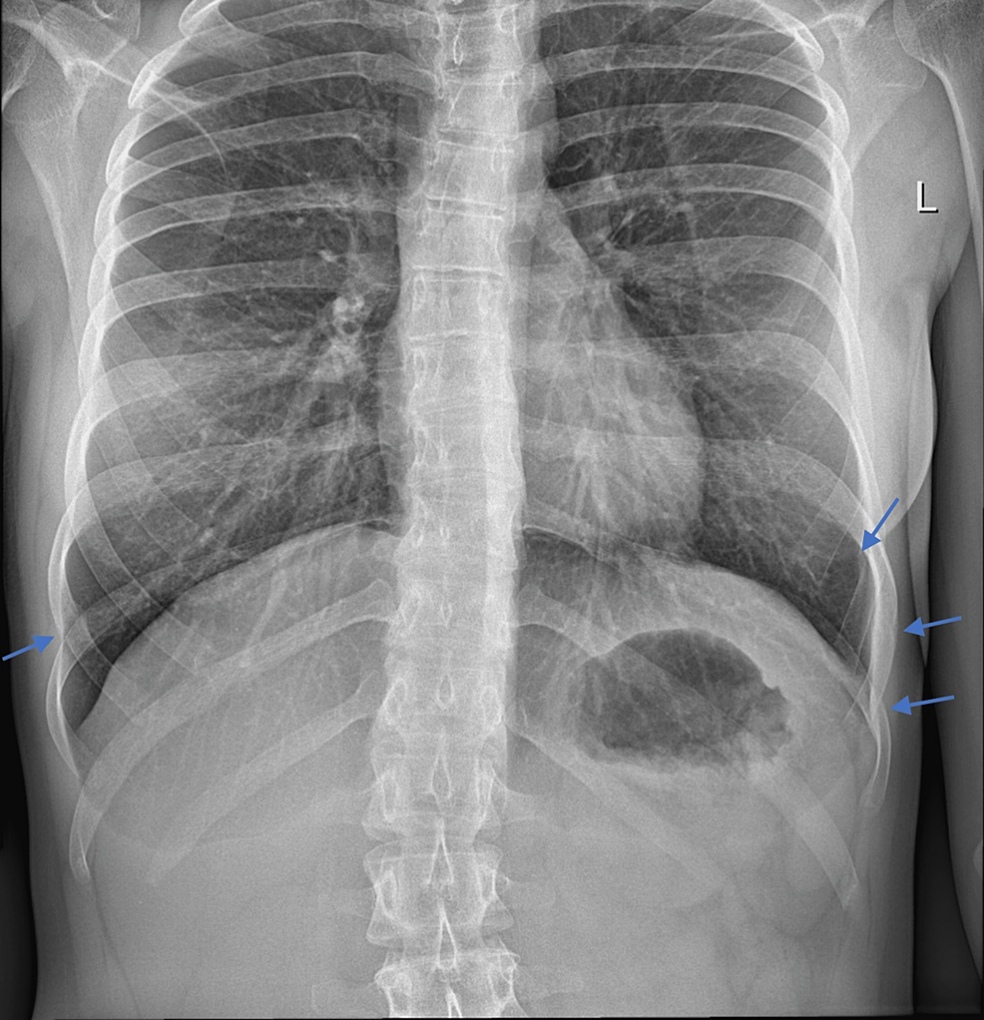
Chest X-ray performed 14 weeks after the former (Figure 2). Blue arrows point to callus formation at all sites of fracture.

Symptoms slowly subsided with the complete disappearance of respiratory manifestations by the end of the second week, and substantial improvement of the stress fracture-related discomfort by day 30, followed by further favorable resolution and callus formation (Figure 3).

## Discussion

Cough-induced rib fractures, also known as "cough fractures" or "cough-related rib injuries," are relatively uncommon medical findings but clinically significant entities, due to the associated symptomatology and the manifestation of potential underlying disorders of the patient's bone physiology.

Biomechanics of coughing and rib fractures

Coughing involves the coordinated action of various muscle groups, including the diaphragm, intercostal muscles, and accessory respiratory muscles. Opposing forces between the serratus anterior and the external abdominal oblique muscle have been proven to cause deformity and subsequent micro- or, in severe cases, complete fractures at the level of the middle third of ribs five to 12. The serratus anterior muscle acts as a scapula stabilizer by fixing the shoulder blade against the rib cage, therefore allowing the lifting of the ribs in a superolateral direction. Conversely, the external abdominal oblique muscle pulls the ribs inferiorly and medially. When muscular absorption and dissipation of opposing force, as well as the elastic limit of the costal apparatus, are exceeded, disruption of the rib continuity occurs [1,3].

Case studies

Hanak et al. analyzed 54 cases of cough-induced rib fractures, diagnosed over a period of nine years, in a retrospective single-center study. Results of this study indicated the prevalence of cough chronicity (paroxysms present for more than three weeks), chest pain with insidious onset (65%), and laterally located fractures (50%). Furthermore, postmenopausal women displayed higher rates of occurrence, which highlighted differences between genders in bone densities, as demonstrated by densitometric investigations [4]. In the case series described by Sano et al., right-sided, single fractures of the middle and lower ribs were registered prevalently [5].

The particularity of the case presented in this paper consists of the young age of the patient, multiple and bilaterally located rib fractures, with a lack of apparent predisposing factors in regard to her bone physiology, as confirmed by laboratory investigations. Interestingly, a higher number of fractures is identified on the left side, where a scoliotic sinistro-convex thoracic curvature is present, therefore a correlation between these elements could be speculated. Scoliosis is indeed characteristically associated with rib deformities, the so-called “rib hump,” caused by rotation of the vertebral bodies of the affected regions. Nevertheless, as demonstrated by Erkula et al. in a study analyzing the computed tomography (CT) scans of 11 scoliosis surgery candidates, there is no correlation between rib deformity, the magnitude of the Cobb angle of the curvature, and the degree of spinal rotation [6].

Diagnostic workup

Diagnosis of coughed-induced rib fractures relies on non-specific clinical presentation, such as localized chest pain and shortness of breath, due to the inability to inhale deeply, and radiological imaging. Chest roentgenography, despite being a valuable first-step diagnostic tool, yields a relatively low sensitivity for the identification of cough-induced rib fractures, as opposed to chest CT scanning, reportedly, 58% versus 100% sensitivity in the cohort investigated by Hanak et al. [4]. Despite the acknowledged high sensitivity of CT scanning in detecting the fractures left occult by X-ray imaging, it was considered unnecessary to expose our patient to further radiations, as the conservatory approach would have remained the first-line management of choice.

Treatment and management

Treatment options for non-displaced rib fractures consist of conservatory therapies, including adequate pain management and rest. Surgical intervention is considered in case of displaced, multiple rib fractures, flail chest, when the conservatory approach failed, or when complications and concomitant injuries must be addressed intraoperatively. The prognosis for cough-induced rib fractures is generally favorable, with most patients experiencing gradual pain relief and functional improvement over several weeks, without needing further investigations. Close monitoring should be warranted for elderly patients or individuals presenting with underlying pathologies to avoid insidious exacerbations and prevent fracture recurrence [7].

## Conclusions

Cough-induced rib fractures should be considered as a potential diagnosis in young patients with strenuous, persistent cough and localized chest pain, despite the rare character of such occurrence. Taking into account the paucity of studies in this field, future research should aim to investigate a possible correlation between existing rib deformity due to scoliotic curvatures and predisposition to micro- and complete costal fractures. In addition, administration of antitussive agents could be implemented in the therapeutic paradigms of moderate to severe respiratory tract infections in this set of patients, when productive cough is not present and all the other criteria for their utilization are fulfilled.
